# Volatile Composition and Biological Activity of Jordanian Commercial Samples of *R. coriaria* L. Fruits

**DOI:** 10.3390/molecules26185691

**Published:** 2021-09-20

**Authors:** Rajashri R. Naik, Ashok K. Shakya, Benedetta Ferri, Ghaleb A. Oriquat, Luisa Pistelli, Nawfal A. M. Numan

**Affiliations:** 1 Department of Pharmaceutical Sciences, Faculty of Pharmacy, Al-Ahliyya Amman University, Amman 19328, Jordan; rsharry@ammanu.edu.jo; 2 Pharmacological & Diagnostic Research Center, Faculty of Pharmacy, Al-Ahliyya Amman University, Amman 19328, Jordan; n.numan@ammanu.edu.jo; 3 Dipartimento di Farmacia, University of Pisa, Via Bonanno 6, 56126 Pisa, Italy; ferribenedetta@live.it (B.F.); luisa.pistelli@unipi.it (L.P.); 4 Faculty of Allied Medical Sciences, Al-Ahliyya Amman University, Amman 19328, Jordan; goreqat@ammanu.edu.jo

**Keywords:** antimicrobial activity, HS-SPME/GC-MS, sumac, antioxidant, DPPH, β-carotene bleaching (BCB) assay, principal component analysis (PCA), hierarchical cluster analysis (HCA)

## Abstract

The present paper reports the GC-HS-SPME analysis of volatile emission and GC-MS analysis of chemical composition of essential oil of *R. coriaria* fruits of eight different samples of *R. coriaria* L. fruits (“sumac” folk name), collected from Jordanian agricultural field and the local market. The analyses show an important variability among the Sumac samples probably due to the origin, cultivation, harvesting period, drying, and conservation of the plant material. The main class of component present in all samples was monoterpenes (43.1 to 72.9%), except for one sample which evidenced a high percentage of sesquiterpene hydrocarbons (38.5%). The oxygenated monoterpenes provided a contribution to total class of monoterpenes ranging from 10.1 to 24.3%. A few samples were rich in monoterpene hydrocarbons. Regarding the single components present in all the volatile emissions, β-caryophyllene was the main compound in most of the analyzed samples, varying from 34.6% to 7.9%. Only two samples were characterized by α-pinene as the main constituent (42.2 and 40.8% respectively). Essential oils were collected using hydro-distillation method. Furfural was the main constituent in almost all the analyzed EOs (4.9 to 48.1%), except in one of them, where β-caryophyllene was the most abundant one. β-caryophyllene ranged from 1.2 to 10.6%. Oxygenated monoterpenes like carvone and carvacrol ranged from 3.2–9.1% and 1.0–7.7% respectively. Cembrene was present in good amount in EO samples EO-2 to EO-8. The antioxidant capacities of the fruit essential oils from *R. coriaria* were assessed using spectrophotometry to measure free radical scavenger 2,2-diphenyl-1-picrylhydrazyl (DPPH) radical and inhibition of β-carotene bleaching (BCB). The essential oils from the fruits of the different samples of *R. coriaria* exhibited the MIC value ranging from 32.8 to 131.25 µg/mL against *S. aureus ATCC 6538* and 131.25 to 262.5 µg/mL against *E. coli ATCC 8739*. The MIC values of ciprofloxacin were 0.59 and 2.34 µg/mL against *S. aureus ATCC 6538* and *E. coli ATCC 8739*, respectively.

## 1. Introduction

Plants belonging to *Rhus* genus are commonly known as sumac. The genus *Rhus* contains around 250 species of angiosperms that belong to the family Anacardiaceae [1]. The different species of the *Rhus* genus are used by the local people for medicinal and traditional purposes, such as *R. glabra* (smooth sumac) which is used as an antibacterial agent in several diseases like syphilis, gonorrhea, dysentery, and gangrene in North America [2]. In the same country the juice prepared from the fruits of *R. typhina* called “Sumac-ade or Rhus juice” serves as a drink and also has medicinal purpose due to its pharmaceutical potentials as an anti-hemorrhoidal, antiseptic, diuretic, stomachic, and tonic [3,4]. *R. coriaria* (sumac) is native to Mediterranean area and southeastern Anatolia Turkey and grown wild in region extending from the Canary Island over the Mediterranean coast line to Iran and Afghanistan [5,6,7,8]. Since 1970s, Sumac was cultivated in Italy, Spain, Turkey, and some Mediterranean Arabic countries. R. coriaria dried fruits, powdered with salt, are used as condiments in the Middle East and Mediterranean cuisine; they are used in salads or sprinkled over the kababs [6] due to their sour taste. In the Middle Eastern countries form Jordan to Egypt, *R. coriaria* is used as a spice in grilled meat, stews, rice, vegetable dishes, and salads. In Jordan, *R. coriaria* is used in traditional medicine for reduction of cholesterol, sweating, and to treat diarrhea. Earlier reports on the biological activity of *R. coriaria* suggest antimicrobial, antifungal, antiviral [9], antioxidant [10,11,12], anti-inflammatory [13], hepatoprotective [14], XO inhibitor [11], hypoglycemic [14,15], and cardiovascular protective properties [16]. Several earlier reports have shown the presence of various biologically active compounds including hydrolysable tannins, large variety of organic acids such as malic acid and citric acid [17,18], fatty acids, vitamins flavonoids, derivatives of terpenoids [19], and among the terpenes the essential oils are rich in monoterpenes and sesquiterpenes [20,21]. Previous studies on the chemical constituents of sumac essential oil from different geographical locations evidenced qualitatively and quantitatively variations in these constituents [17,22,23,24,25,26]. Giovanelli and coworkers reported around 169 terpene and non-terpene compounds in the *R. coriaria* essential oils grown in Italy (Sicilian variety) using HS-SPME/GC-MS [27]. They reported also the presence of marker compounds which are non-terpenes like nonanal and *p*-anisaldehyde [17,27], sesquiterpene hydrocarbons such as (*E*)-caryophyllene [23,24] and the diterpene cembrene [24,27]. Recently, Elagbar et al. (2020) reviewed extensively the phytochemical diversity and the pharmacological properties such as antibacterial, antioxidant, hypoglycemic, antimicrobial, antitumor, antiviral, anti-inflammatory, antihepatitis, antiulverigenic, cardioprotective, anticholinesterase, anticancer, anticonvulsant, hepatoprotective, and neuroprotective activities of *R. coriaria* and other *Rhus* species [28]. Farag et al. (2018) reported 74 volatile emissions in *R. coriaria* fruits (sumac) and roasted fruits from three different geographical countries (Egypt, Palestine, and Jordan) using the solid phase microextraction (HS-SPME) [29]. 

*R. coriaria* L. is also used as a herbal remedy in traditional medicine due to its analgesic, antidiarrheic, antiseptic, anorexic, and anti-hyperglycemic properties [9]. However, the extract of *R. coriaria*, which protects humans against oxidative DNA-damage [30], is most notable for its antimicrobial and antioxidant activities [7,10,11,31,32,33,34]. The commercial samples of sumac are processed using water for culinary purpose. 

No previous studies on the commercial samples of sumac are available in the literature till now in depth and the aim of the present study is to evidence the difference among the different samples cultivated or collected in the different area of Jordan. This paper describes the HS-SPME-GC-MS analysis of volatile emission and GC-MS analysis of chemical composition of essential oil of *R. coriaria* fruits collected from agricultural fields and commercial samples from different regions of Jordan, in order to identify the phytochemicals present for pharmaceutical purposes, industrial uses, and mainly as food supplements. 

## 2. Results

The main class of components present in the volatiles spontaneously emitted was represented by total monoterpenes (from 43.1% in sample 6 to 72.9% in sample 2) except for the sample 1 where a high percentage of sesquiterpene hydrocarbons was evidenced (38.5%) (Supporting Data Table S1). The monoterpene hydrocarbons showed a higher amount among the total monoterpenes especially in samples 2 and 8, with a total absence in sample 1. Oxygenated monoterpenes provided a less contribution to the total class of monoterpenes ranging from 10.1 to 24.3%, except in the sample 8 where they showed a minimal percentage of 3.3%. Sesquiterpene hydrocarbons predominated in all the analyzed samples (from 11.8 to 38.5%) while oxygenated sesquiterpenes were present with percentages lower than 1%. These latter compounds were totally absent in samples 1 and 2. 

Non-terpenes were the main components only in sample 1 (40.7%) even though they were present in all the sumac samples with lower percentages (from 5.6% in 5 and 16.6% in sample 6). The high percentage of phenylpropanoids (10.1%) in sample 5 must be emphasized. These results were partially in agreement with that reported by Giovanelli et al. [3,4] who reported the volatiles from Italian wild sumac; in fact monoterpenes dominated in the half of that samples and non-terpenes in the others. However, in the aroma profile of the Jordanian sample, purchased from local market in 2014, and reported in that work, a similar composition was evidenced with the samples analyzed herein. α-Pinene and β-caryophyllene predominated in both works.

Regarding the single components present in all the volatile emissions, β-caryophyllene was the main compound in most of the analyzed samples, varying from 34.6% in 1 to 7.9% in 2. Samples 2 and 8 were characterized by α-pinene as the main constituents (42.2% and 40.8 respectively). Sample 1 showed a different behavior from the rest of the samples, since it evidenced high amounts of non-terpenic components such as: *n*-nonanal > dodecanol > decanal > dodecanal > pentadecane > tetradecanal > undecanal > tridecane. Among these non-terpenes, the *n*-nonanal showed the highest percentage (10.6%) even though it was present in all the other samples with considerably lower percentages, followed by decanal (5.71% in 1) if compared with the lower percentages (less than 1%) in all other samples. Pentadecane, dodecanol, dodecanal, and undecanal were exclusive components in sample 1. On the contrary, the sample 6 was characterized by the exclusive presence of these compounds: 2-pentylfuran > methyl chavicol > (*E*)-2-hexenyl acetate > thymoquinone > β-gurjunene > α-phellandrene > eugenol > 4-vinyl guaiacol > α-guaiene (4.1 > 2.8 > 1.2 > 0.8 > 0.7 > 0.7 > 0.6 > 0.4 > 0.2%, respectively). The β-caryophyllene content was higher in the Jordanian samples than in the results reported by Farag et al. [3,4]. 

**Figure 1:**
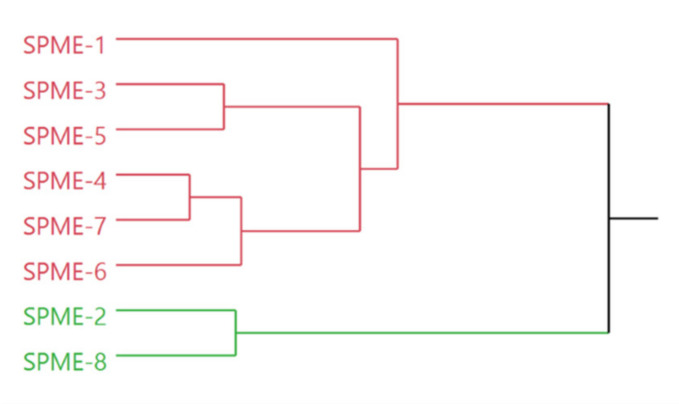
Dendrogram of the *Rhus coriaria* samples resulting from the HS-SPME analysis.

The dendrogram obtained by the HCA carried out on the complete composition of the headspaces (Figure 1) evidenced a different grouping behavior of the studied samples compared to the HCA performed on the EOs chemical composition. 

Moreover, in this case, the samples were divided into two macro-clusters: the first one, highlighted in pink, contained six units, while the second one, evidenced in green contained only two sample, SPME-2 and SPME-8. The pink cluster, in turn, presented two internal groups, the first represented only by SPME-1 and the latter characterized by two sub-groups, one comprising SPME-3 and SPME-5, and the other SPME-4, SPME-7, and SPME-6. The SPME-2 sample was collected from Ajloun, Jordan which is located at higher altitude of ~1100 m than the other areas. Marketed samples 1, 3, 4, 5, and 8 were collected from the different commercial shops.

**Figure 2:**
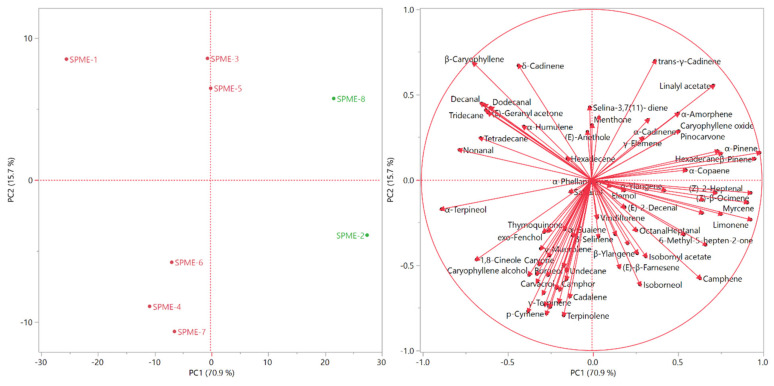
The score and the loading plot of the principal component analysis of Sumac volatile samples.

The score and the loading plots of the PCA performed on the headspaces chemical composition are reported in Figure 2.

The positioning of the samples in the score plot overlapped the clusterization of the HCA. The samples of the green cluster were plotted in the rightmost area of the right quadrants (PC1 > 0): SPME-8 in the upper quadrant (PC2 > 0), and SPME-2 in the bottom one (PC2 < 0). The samples of the pink cluster, as in the HCA, formed three internal groups: SPME-1 was plotted alone in the upper left quadrant (PC1 < 0; PC2 > 0), SPME-3 and SPME-5 were located on the partition line between the upper left and upper right quadrants (PC2 > 0), and finally SPME-4, SPME-7, and SPME-6 were thrown in the bottom left quadrant (PC1 < 0; PC2 < 0). Commercial sample SPME-4 clustered with the SPME-6 and SPME-7 which are collected from the Al-Salt and Al-Fuheis agricultural field have the same altitude and weather conditions.

GC-MS analysis of the essential oils from *R. coriaria* fruits (Sumac EO samples 1–8) are reported in Supporting Data Table S2. It is important to mention that the percentage of essential oils was very less ranging from 0.18 to 0.31%. The analyses showed an important variability among the Sumac samples probably due to the origin, cultivation, harvesting period, drying, and conservation of the plant material. The fresh sumac sample 2, sample-6, and sample-7 were collected from the Ajloun Forest Reserve, Al-Salt and Al-Fuheis agricultural field respectively, which are located between 800 and 1100 m of altitude. Commercial samples were collected from Jordanian local market. The essential oils were hydrodistilled using a Clavenger apparatus and collected with *n*-hexane. The total identified compounds ranged from 89.3 to 98.8%. 

The majority of the identified compounds were non-terpenes with the higher relative amount in EO-1 (73.2%) and EO-3 (68.8%). Furfural was the main constituent whose percentage was minimal in EO-5 (4.9%) and maximum in EO-3 (48.1%). The marketed sumac fruits contained volatiles along with some fixed oil. Palmitic acid was high in samples EO-5 and EO-2 (9.4% and 9.0%) followed by EO-4 and EO-6 with 8.8% and 6.6%, respectively. It was not detected in sample EO-1. Among the other non-terpene compounds the percentage of methyl elaidate was consistent in samples EO-3, EO-4, and EO-5 (4.4, 4.2, and 4.7%, respectively), absent in EO-1; the same for methyl palmitate from 1.3% in EO-8 to 3.4% in EO-5, except in EO-1. Furthermore, oleic acid was evidenced in good amount in four of the eight samples, especially in EO-2 (5.8%) and EO-5 (4.4%). Noteworthy is the presence of (*E*)–cinnamaldehyde in EO-1 (8.5%), followed by EO-6 with 2.6% and EO-3 which contained 0.8%. The rest of the samples had zero contribution. (*E*)–2-Heptenal was present in all the samples, very high in EO-1 (5.1%) while the amount in the other sample ranged from 0.1 to 1.3%.

The total monoterpenes ranged from 9.3% in EO-7 to 19.0% EO-5, which mainly consisted of oxygenated monoterpenes, while the monoterpene hydrocarbons showed very low percentages (from 0.1% in EO-4 to 2.5% in EO-3, respectively). The main compounds in the oxygenated monoterpenes were represented by carvone (9.1% in EO-5 followed by EO-4 and EO-2 with 7.8% and 7.2%, respectively), carvacrol (from 1.0% in EO-7 to 7.7 and 6.9 % in EO-1 and 2, respectively), α-Terpineol (ranged from 1.4% to 3.0%), and 4-terpineol (only absent in EO-8). The Sicilian variety of sumac contained transient amount of carvacrol and carvone [27]. 

The sesquiterpenes varied from 2.0% in EO-1 to 25.3% in EO-7, mainly due to sesquiterpene hydrocarbons, like β-caryophyllene present in all the samples even though with different percentages; in fact, it was high in samples EO-7 and EO-5 (10.6% and 10.1%) followed by sample EO-4 and EO-8 (8.7% and 8.1%, respectively) and EO-2 with 7.5%. Only in sample EO-1 the percentage of β-caryophyllene decreased till 1.2%. Moreover, α-humulene, β–cadinene, and δ–cadinene were present in all samples in low percentages, except in sample EO-1 and sample EO-8 (only for δ–cadinene). The amount of β-caryophyllene in EOs was comparable to Italian (Sicilian variety) samples [27] and Turkish samples [22]. Samples analyzed in this research work shows low level of β-caryophyllene as compared to data reported earlier [27]. Bahar and Altug reported the good amount of β-caryophyllene (28.5%) and malic acid (13.9%) in Turkish sample of Sumach [22].

Caryophyllene alcohol was the only representative compound of the oxygenated sesquiterpenes, ranging from 0.4% in EO-3 to 1.0% in both EO-5 and EO-7. Another substance evidenced in all the EO samples was the diterpene cembrene with the highest relative percentage in sample EO-7 (10.7%) followed by sample EO-5 and EO-3 (8.4% and 8.0%, respectively) up to 0.2% in sample EO-1. The level of cembrene in EO of Sumac was similar to the Italian samples (Sicilian sample) [27].

Interesting to note the relative amount of phenyl-propanoid as (*E*)-Anethole in sample EO-1 (4.1%) together with the percentages almost consistent in sample EO-4, EO-5, and EO-6 (3.4%, 3.9%, and 3.1%, respectively). However, the same compound was undetected in samples EO-2, EO-3, and EO-8. Eugenol in sample 1 was the highest evidenced (2.9%) together with the 1.5% of sample EO-6, while it was totally absent in samples EO-6 and EO-7. 

The chemical composition of the hydro-distilled essential oil of sumac has been investigated in different countries including Iran, Turkey, and Italy. The chemical composition of the hydro-distilled essential oils extracted from *R. coriaria* fruits collected from 14 different locations in Iran was studied [35]. The results obtained from this investigation, based on principle component analysis (PCA) and cluster analysis (CA) revealed at least five different chemotypes. These included the (*E*)-caryophyllene, (*E*)-caryophyllene/α-pinene, (*E*)-caryophyllene/cembrene, nonanoic acid/cembrene, *n*-nonanal/(*2E*,*4E*)-decadienal. None of these chemotypes matched the samples analyzed herein. Only EO-5 showed good percentages of β-caryophyllene (10.1%) and cembrene (8.4%) followed by palmitic acid (9.4%) and carvone (9.1%). It is also well reported that the β-caroyophyllene, cembrene, *n*-nonanal, and *p*-anisaldehyde were the major compounds too [28], but not in our samples.

It is well-known that the *Rhus coriaria* fruits showed variations in the chemical composition of the EOs from different locations which might be due to the geographical distribution, environmental factor, harvesting time, extraction, and analytical methods used. Although *R. coriaria* is not recognized as an aromatic plant, its fruit is enriched in essential oil composed of monoterpenes and/or sesquiterpenes [22,27].

**Figure 3:**
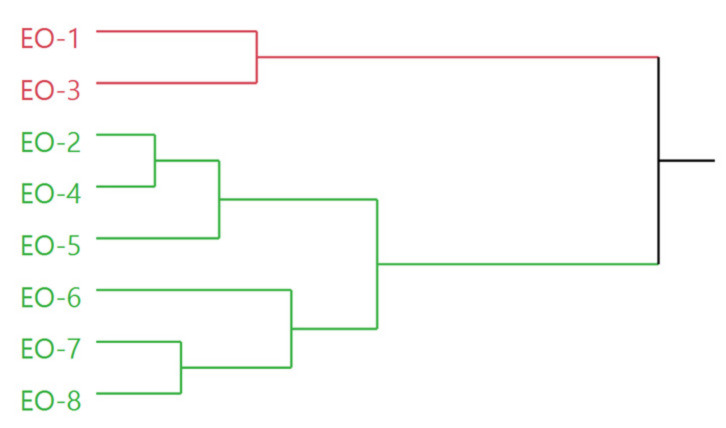
Dendrogram of the *Rhus coriaria* samples resulting from the EO analysis.

The dendrogram obtained by the hierarchical cluster analysis (HCA) performed on the complete composition of the EOs obtained from the analyzed samples (Figure 3) evidenced a partition of the samples in two macro-clusters. The pink cluster was formed by only two samples, EO-1 and EO-3, whereas the green cluster presented six samples, divided in turn into two internal groups, one comprising EO-2, EO-4, and EO-5, and the other EO-6, EO-7, and EO-8.

**Figure 4:**
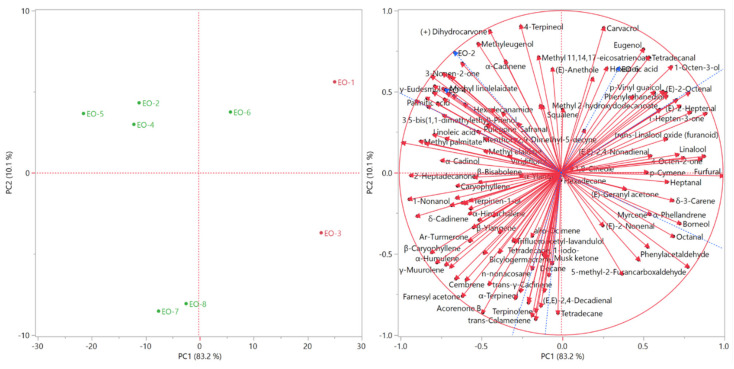
The score and the loading plot of the principal component analysis of all the EOs obtained.

The score and the loading plot of the principal component analysis are reported in Figure 4. The distribution of the samples in the score plot was comparable to that of the HCA. EO-1 and EO-3, belonging to the pink cluster, were plotted in the rightmost area of the right quadrants (PC1 > 0), the first in the upper quadrant (PC2 > 0), and the latter in the bottom one (PC2 < 0). The samples of the green cluster occupied almost the whole remaining part of the plot, while maintaining the internal partition evidenced by the HCA. The samples EO-2, EO-4, and EO-5, indeed, were plotted in the upper left quadrant (PC1 < 0; PC2 > 0), EO-7 and EO-8 were positioned in the bottom left one (PC1 < 0; PC2 < 0), and EO-6 was plotted in the upper right quadrant (PC1 > 0; PC2 > 0), in an intermediate position among the other green samples, although the HCA EO-6 cluster was close to EO-7 and EO-8 which were collected from the Al-Salt and Al-Fuheis city that lie in the same altitude. The commercial samples 1, 3, 4, 5, and 8 that were collected from market does not show cohesiveness in their chemical behavior as they might have originated from different altitudes or locations.

### 2.1. The Antioxidant Activity

The results of the antioxidant activity of *R. coriaria* essential oils for DPPH free radical scavenging and β-carotene bleaching (BCB) assay are represented in the Table 1. The samples of essential oil exhibited significant free radical scavenging activity against DPPH free radical, and the IC_50_ value of different samples of the essential oil (sample 1–8) ranging from 128.5 to 259.1 µg/mL respectively, these results were compared with ascorbic acid (a standard antioxidant) having IC_50_ concentration of 4.05 µg/mL. The different essential oil samples (1–8) against the β-carotene bleaching (BCB) assay showed promising results. The mean IC_50_ values of samples (1–8) were 175.2, 310.5, 169.5, 221.5, 165.5, 195.0, 225.0, and 165.5 µg/mL respectively, which were compared with the standard antioxidant rutin (IC_50_ 2.93 µg/mL). 

The potential antioxidant activity exhibited by the essential oil may be due to the presence of a variety of chemical compounds like β-caryophyllene [36,37], (E)-cinnamaldehyde [38], carvacrol [39], carvone [40], cembrene [41], eugenol [42], methyl palmitate, palmitic acid, oleic acid [28,43], α-copaene [44], α-himachalene [45], α-humulene [46], α-terpineol [47], and δ-cadinene [48] which are present in the these samples. All these compounds are collectively responsible for antioxidant activity.

### 2.2. Antibacterial Activity

The antimicrobial activity and the minimum inhibitory concentration (MIC) were determined on *S. aureus ATCC 6538*, and *E. coli ATCC 8739* using the reported procedure. The essential oils from the fruit of different samples of *R. coriaria* L. exhibited the MIC value ranging from 32.8 to 262.5. µg/mL compared to ciprofloxacin, 0.59 to 2.34 µg/mL (Table 2) against *S. aureus ATCC 6538*, and *E. coli ATCC 8739*. The essential oil samples EO-5 and EO-8 were more active (MIC 32.80 µg/mL) against *S. aureus* ATCC 6538 than the essential oil samples sample number EO-1, EO-3, EO-6, and EO-7 (MIC 32.80 µg/mL). The sample EO-2 and EO-4 have MIC value of 131.25 µg/mL. The essential oil samples EO-1, EO-3, EO-5, and EO-8 showed their activities with a MIC of 131.25 µg/mL against Gram-negative microorganism, while essential oil EO-2 and EO-4 showed the similar effect at 262.50 µg/mL. The essential oils were more effective against the Gram-positive strain than the Gram negative; it might be due to the presence of more lipophilic constituents in the essential oils. 

The strong antimicrobial activity of these essential oils might be due to synergistic effects of chemical constituent (both lipophilic and hydrophilic) present in the EOs, like β-caryophyllene [36,37], carvacrol [49], carvone [50], cembrene [51], eugenol [52,53], γ-terpinene, 1,8-cineole, α-terpinene [54], α-terpineol [55], α-Himachalene, α-Humulene [37], and (E)-Anethol [53].

## 3. Materials and Methods

Commercial samples of *R. coriaria* L. fruits in the full ripe stage were purchased from local suppliers and from the commercial shops (at Amman, Al-Fuheis, Al-Salt and Ajloun, Jordan, Table 1 and Table 2) during July-August (2017–2018). Voucher specimen samples were kept at the Faculty of Pharmacy and Medical Sciences, Al-Ahliyya Amman University, Amman, Jordan. The fruits were stored at −20 °C till further analysis. Samples were also collected from the wild agricultural field at Al-Fuheis (Altitude ~850 m), Al-Salt (Altitude ~800 m) and Ajloun Forest reserve (Altitude ~900 to 1100 m), Ajloun Jordan (Tables S1 and S2) and dried. All the fruit samples were analyzed using HS-SPME/GC-MS at the University of Pisa, Italy and the essential oil samples were analyzed using GC-MS at Al-Ahliyya Amman University, Amman.

### 3.1. Chemicals and Materials

Supelco SPME device (Supelco Analytical, Bellefonte, PA, USA), SPME fiber of Stableflex-coated PDMS (polydimethylsiloxane) (57302) were purchased by Supelco (Oakville, ON, Canada) through a local supplier. All the other chemicals and standards were purchased from Sigma Aldrich (St. Louis, MO, USA).

### 3.2. Essential Oil Extraction

Essential oils from the different fruit samples of powdered Sumac were obtained using a Clevenger type apparatus as reported earlier [56], using 100 g of each sample. The essential oil amount was very low (0.18 to 0.31%). Hence, the essential oil samples were collected and recovered using *n*-hexane. EO was collected and dried over anhydrous sodium sulfate. The EOs were stored at −20 °C under nitrogen till further analysis.

### 3.3. HS-SPME of Volatile Compounds

Fruits of each sample were analyzed for their spontaneous volatile emission by HS-SPME. Total of 1 g (about 20 fruit) of each sample was introduced into a 25-mL glass conical flask closed with aluminum foil and allowed to equilibrate for 30 min. After the equilibration time, a Supelco SPME device (Supelco Analytical, Bellefonte, PA, USA), coated with polydimethylsiloxane (PDMS, 100 µm), was inserted through the septum. Then the fiber was exposed to the headspace of the vial for 30 min at room temperature. Once sampling was finished, the fiber was withdrawn into the needle and transferred to the injection port of the GC–MS system. All the SPME sampling and desorption conditions were identical for all the samples. 

### 3.4. GC-FID and GC-MS Analysis

These analyses were performed according to the method previously described [27,57]. The GC-FID analysis was accomplished with an HP-5890 Series II instrument equipped with a HP-Wax and DB-5 capillary columns (both 30 m × 0.25 mm, 0.25 µm film thickness), working with the following temperature program: 60 °C for 10 min, rising at 5 °C/min to 220 °C. The injector and detector temperatures were maintained at 250 °C; carrier gas, nitrogen (2 mL/min); split less injection. GC-MS analyses were carried out with a Varian CP3800 gas chromatograph equipped with a DB-5 capillary column (30 m × 0.25 mm; coating thickness, 0.25 µm) and a Varian Saturn 2000 ion trap mass detector. Analytical conditions: injector and transfer line temperature, 220 and 240 °C at 3 °C, respectively; oven temperature, programmed from 60 to 240 °C at 3 °C; carrier gas, helium (1 mL/min). Samples were desorbed from the SPME fiber as a split-less injection mode for one minute.

### 3.5. GC-MS Analysis of EOs

Analysis of EOs was carried out using Shimadzu QP2020 GC-MS equipped with a split-split-less injector, DB5 MS fused silica column (5% phenyl, 95% polydimethylsiloxane 30 m × 0.25 mm, film thickness 0.25 µm). A linear temperature program was used to separate the EOs components. Temperature programming was applied at 7 °C/min heating rate starting from 50 °C (initial temperature) to 280 °C (final temperature) and held at 280 °C for 40 min with a total run time of 74 min. Injector temperature was 260 °C with a split ratio of 20:1; injection volume (1 µL, 4% solution in DCM); carrier gas: helium; MS source temperature/detector temperature 240 °C; interface temperature 250 °C; ionization energy (EI) 70 eV; and AMU gain −492; AMU offs −67; ionization current 60 µm; scan range 35–500 amu; scan speed 1666. Solvent cut was 3 min; while the data were acquired from 4.5 min. Mass spectrum of every chemical constituent was compared with corresponding reported spectrum (in NIST 2017, and ADAMS-2007 libraries) for GC-MS and published references. Identification of compounds was also confirmed by comparing their relative retention indices (RRI) relative to n-alkanes (C_8_−C_35_) with reported values in the literature including Adam’s library.

### 3.6. Identification of Compounds

The identification of the constituents was based on the comparison of their retention time (Rt) with those of pure authentic samples, comparing their linear indices (LRI) relative to a series of *n*-hydrocarbons, and on computer matching against commercial [58,59], and also made possible by the use of a homemade library of mass spectra built up from pure substances and components of known oils, and MS literature data. 

### 3.7. Multivariate Statistical Analysis

The multivariate statistical analysis was carried out with the JMP Pro 13.0.0 software package (SAS Institute, Cary, NC, USA).

Data used for the statistical analysis of the EOs and the headspace compositions were 165 × 8 (165 individual compounds × 8 samples = 1320 data) and 95 × 8 (95 individual compounds × 8 samples = 760 data), respectively. The principal component analysis (PCA) was performed selecting the two highest principal components (PCs) obtained by the linear regressions operated on mean-centered, unscaled data: this analysis aimed at reducing the dimensionality of the multivariate data of the matrix, preserving most of the variance. For the EOs chemical composition, the chosen PC1 and PC2 covered 83.2% and 10.1% of the variance, respectively, for a total explained variance of 93.3%, while for the complete headspace compositions, the selected PC1 and PC2 studied 70.9% and 15.7% of the variance, for a total of 86.6%. The hierarchical cluster analysis (HCA) were performed using Ward’s method, with squared Euclidean distances as a measure of similarity. Both the HCA and the PCA methods can be applied to observe the groups of samples even when there are no reference samples that can be used as a training set to establish the model.

### 3.8. Antioxidant Activity

#### DPPH (2-Diphenyl-1-picryl-hydrazyl) Free Radical Scavenging Activity

The radical scavenging activity of the essential oil was evaluated using reported method with slight modification. A stock solution of DPPH (0.002% *w*/*v*) was prepared in methanol. Different methanolic concentrations of the EO samples (1.56–100 μg/mL) were prepared as serial dilution method. About 200 μL of DPPH solution is mixed with 100 μL sample from different concentrations in a 96-well ELISA plate. These plates were incubated in (Synergy HTX Multi-Mode Reader, Biotek, Winooski, VT, USA) dark for 30 min. Synergy HTX Multi-Mode Reader was used for measuring the absorbance of color at 517 nm. Results were compared with different concentrations (0.2–25 μg/mL) of ascorbic acid (standard). DPPH radical scavenging activity was determined and the IC_50_ was calculated using Sigma-Plot ver. 9

(1) $$\%\text{ Free radical Scavenging activity }=\frac{\left(\text{Absorbance of control }-\text{ Absorbance of sample}\right)}{\text{ Absorbance of control.}}\times 100.$$

### 3.9. β-Carotene Bleaching (BCB) Assay

A solution of β-carotene/linolenic acid was prepared by dissolving β-carotene (5 mg) in 50 mL of chloroform. In a separate Erlenmeyer flask, 40 mg linoleic acid and 400 mg Tween-40 were taken and an aliquot of β-carotene (3 mL) solution was added. It was mixed and set aside for 2 min. The chloroform was evaporated off using nitrogen (N2) gas. About 100 mL distilled water was added to the mixture. Immediately after preparation, the absorbance of this solution was recorded at 470 and 700 nm. Total of 3 mL aliquots of β-carotene/linoleic acid emulsion was mixed with different concentrations of the essential oil (1 µg/mL to 512 µg/mL). The test and control (containing water in place of sample) tubes were capped and incubated at 50 °C. The absorbance of the emulsion at λ_470_ and λ_700_ nm was determined after 1 h. All determinations were carried out in triplicate. The degradation rate and antioxidant activities were calculated using the formula:

(2) Degradation rate (DR) of beta carotene=LinA intialA sample60

(3) $$\text{Antioxidant activity (\%)}=\frac{\left(\text{degradation rate of control }-\text{degradation rate of sample}\right)}{\text{degradation rate of control}}\times 100$$

### 3.10. Antibacterial Activity

The antibacterial activity of the oil was evaluated by agar diffusion method [60] against two bacterial species Gram negative strains Escherichia coli ATCC 8739 and Gram positive strain, Staphylococcus aureus ATCC 6538. Wells of 6-mm diameter were dug on the inoculated nutrient agar medium with sterile Biopsy punch and 50 μL of sample in 3.0% DMSO (dimethyl sulfoxide) were added in each well. The plates were incubated at 37.0 ± 0.5 °C for 24 to 48 h. Plates were removed from the incubator and the diameter of zone of inhibition was measured in mm.

In a separate experiment (using broth microdilution method), the minimum inhibitory concentration (MIC_50_) was determined according to the National Committee for Clinical Laboratory Standards (NCCLS) with some modification. MIC tests were performed in 96 flat bottom micro-titer plates (TPP, Switzerland) as reported earlier [60]. Different concentration of samples (350.0 to 2.73 μg/mL) were prepared using the serial dilution method and evaluated for the activity. The standard solutions of ciprofloxacin (50 to 0.39 μg/mL) were prepared using serial dilution method.

In all assays, controls (negative as well as positive) were prepared. Negative control for 3.0% DMSO was carried out to check its activity. MICs were expressed as the average of two successive concentrations of the antimicrobial agent showing no growth and growth, respectively. The growth of microorganism was detected as turbidity, using a Synergy HTX Multi-Mode Reader, Biotek, USA (at 630 nm) comparing to the turbidity of an un-inoculated well.

## 4. Conclusions

The present study indicates that the samples of sumac marketed in Jordan have significant antioxidant and anti-microbial activities. The antioxidant and antibacterial activities are due to the various terpenoids and polyphenols present in the oil. Currently there is considerable interest in new natural antioxidants and antibacterial agents to replace the synthetic ones that are used in foods and pharmaceutical industries. The phenols together with other chemical constituents, which are present in the fruits, might be responsible for the synergistic activity. This work can contribute to the knowledge of the substances present in the aroma profile of this spice (Sumac) used in Jordan as an essential condiment and cultivated and processed under the supervision of the small industry guideline.

## Figures and Tables

**Table 1:** DPPH radical scavenging activity and β-carotene bleaching (BCB) inhibition activity of *R. coriaria* L. essential oil (Sumac EOs 1–8) from the Jordanian market and agricultural field.

Antioxidant Activity ^a^	Sample ^#^									
	Ascorbic Acid	Rutin	EO-1	EO-2	EO-3	EO-4	EO-5	EO-6	EO-7	EO-8
DPPH radical scavenging activity	4.05	-	128.5	259.1	140.1	160.9	135.8	175.4	205.3	145.5
β-carotene bleaching (BCB) assay	-	2.93	175.2	310.5	169.5	221.5	165.5	195.0	225.0	165.1

^a^ Activities of EO samples are expressed as IC_50_ (µg/mL); ^#^ samples of Sumac were collected from the different location and local market from Jordan. Sample 1 (Amman, Downtown), Sample 3 (Amman City), Sample 4 (city Mall), Sample 5 (Al-Fuheis), and Sample 8 (Al-salt City). Sample 2 (Ajloun Forest Reserve), Sample 6 (Al-Salt field), and Sample 7 (Al-Fuheis field).

**Table 2:** Antibacterial activity of *R. coriaria* L essential oils (Sumac EO, 1–8) from the Jordanian market and agricultural field.

Microorganism	Sample ^#^									
	3.0% DMSO	Ciprofloxacin	EO-1	EO-2	EO-3	EO-4	EO-5	EO-6	EO-7	EO-8
	**Zone of Inhibition (mm) ^a^**									
*Staphylococcus aureus* ATCC 6538	-	24	12	10	13	13	14	13	11	15
*E. coli ATCC 8739*	-	18	9	8	10	9	11	9	8	11
	**Minimum Inhibitory Concentration ^b^ (µg/mL)**									
*Staphylococcus aureus* ATCC 6538	-	0.59	65.80	131.25	65.80	131.25	32.80	65.80	65.80	32.80
*E. coli* ATCC 8739	-	2.34	131.25	262.50	131.25	262.50	131.25	131.25	131.25	131.25

Diffusion method. ^a^ Diameter of well = 6 mm, n = 2, ^b^ Minimum inhibitory concentration, n = 2; - = no inhibition in microbial growth. ^#^ Samples of Sumac were collected from the different location and local market from Jordan. Sample 1 (Amman, Downtown), Sample 3 (Amman City), Sample 4 (city Mall), Sample 5 (Al-Fuheis), and Sample 8 (Al-salt City). Sample 2 (Ajloun Forest Reserve), Sample 6 (Al-Salt field), and Sample 7 (Al-Fuheis field).

## Data Availability

Data sharing not applicable.

## Supplementary Materials

The following are available online, Table S1: Volatile composition of *R. coriaria* fruits (Sumac samples 1–8) purchased from the Jordanian market and agriculture field. Table S2: GC-MS analysis of Essential oil of *R. coriaria* fruits (Sumac samples 1–8) purchased from the Jordanian market and agriculture field.

## Sample Availability

Samples are available from the communicating author.
