# Supplementary material for: Volatile Composition and Biological Activity of Jordanian Commercial Samples of R. coriaria L. Fruits
Source: Molecules. 2021 Sep 20;26(18):5691. doi: 10.3390/molecules26185691 (PMC8469490; doi:10.3390/molecules26185691)
Supplement: Supplementary file 1 [file molecules-26-05691-s001.zip › molecules-1355474-supplementary.pdf]

Supplementary Materials

Table S1. Volatile composition of *R. coriaria* fruits (Sumac samples 1-8) purchased from the Jordanian market and agriculture field.

N°	Class	Compound	LRI [a]	Samples (relative content, %) [#]							
				**1**	**2**	**3**	**4**	**5**	**6**	**7**	**8**
1	nt	Hexanal	806	-	0.7	0.1	0.3	0.3	1.0	0.2	1.3
2	nt	(*E*)-2-Hexenal	854	-	0.1	-	-	-	-	-	-
3	nt	Heptanal	901	-	0.2	-	-	-	0.1	-	-
4	mh	Tricyclene	930	-	0.2	-	-	-	-	-	-
5	mh	**α-Pinene**	**939**	**-**	**42.2**	**22.2**	**8.2**	**20.1**	**10.4**	**11.0**	**40.8**
6	mh	Camphene	953	-	2.0	0.7	1.0	0.7	0.8	1.0	0.5
7	nt	(*Z*)-2-Heptenal	960	-	0.6	-	-	0.3	0.4	-	0.3
8	mh	β-Pinene	980	-	3.9	1.3	0.3	1.1	0.8	0.2	2.8
9	nt	1-Octen-3-ol	982	-	-	-	0.3	-	-	0.5	-
10	nt	6-Methyl-5-hepten-2-one	988	-	1.2	0.3	0.5	0.8	1.2	0.5	0.8
11	mh	Myrcene	991	-	2.8	1.3	1.6	1.9	-	1.9	1.9
12	nt	2-Pentyl furan	993	-	-	-	-	-	4.1	-	-
13	nt	Octanal	1002	-	1.0	-	1.1	0.8	-	-	-
14	mh	α-Phellandrene	1005	-	-	0.7	-	-	-	0.9	0.5
15	nt	Butyl pivalate	1007	-	-	-	-	-	0.7	-	-
16	mh	δ-3-Carene	1011	-	-	0.2	0.5	-	-	0.4	-
17	mh	α-Terpinene	1018	-	0.5	0.5	2.4	0.5	-	3.0	0.2
18	nt	(*E*)-2-Hexenyl acetate	1020	-	-	-	-	-	1.2	-	-
19	mh	*p*-Cymene	1027	-	0.5	0.6	5.8	0.4	1.2	8.5	0.4
20	mh	Limonene	1031	-	8.8	4.4	4.9	5.9	3.8	5.1	7.6
21	om	1,8-Cineole	1035	5.2	-	2.6	10.4	-	2.6	5.1	-
22	mh	(Z)-β-Ocimene	1040	-	2.4	0.4	0.3	0.9	0.2	1.0	1.9
23	mh	(E)-β-Ocimene	1050	-	3.8	0.7	0.5	1.3	0.5	1.2	2.0
24	mh	γ-Terpinene	1062	-	0.5	1.0	5.5	1.0	1.0	8.0	0.8
25	nt	(*E*)-2-Octenal	1063	-	0.6	-	-	-	1.0	-	-
26	om	*trans*-Linalool oxide (furanoid)	1076	-	0.6	0.4	0.9	1.3	0.9	0.5	0.2
27	mh	Terpinolene	1088	-	0.6	0.6	1.5	0.9	1.0	1.3	0.2
28	nt	Undecane	1099	-	-	-	-	-	-	0.5	-
29	om	Linalool	1100	-	-	-	0.2	-	1.0	-	0.2
30	nt	Nonanal	1103	10.6	2.0	2.4	4.7	2.2	4.1	3.0	1.7
31	nt	Isopentyl 2-methyl butanoate	1105	-	0.3	0.2	-	-	-	-	0.1
32	om	*exo*-Fenchol	1117	-	-	0.4	0.6	-	-	0.2	-
33	om	Terpinen-1-ol	1138	-	-	-	0.1	-	-	-	-
34	om	*trans*-Pinocarveol	1140	-	-	-		-	-	-	0.3
35	om	Camphor	1145	-	0.2	0.2	0.4	-	0.9	0.3	-
36	om	Menthone	1154	-	-	-	-	1.1	-	-	-
37	om	Isoborneol	1156	-	6.1	0.5	2.3	3.5	5.9	3.2	-
38	om	Pinocarvone	1164	-	-	-	-	-	-	-	0.5
39	om	Borneol	1166	-	0.1	0.6	0.8	-	0.3	0.6	0.1
40	om	Menthol	1173	-	-	0.1	-	2.5			0.2
41	om	4-Terpineol	1180	-	0.2	1.2	1.6	0.4	0.9	1.1	0.1
42	om	α-Terpineol	1190	1.9	0.6	1.8	1.9	1.1	1.9	1.5	0.2
43	pp	Methyl chavicol	1197	-	-	-	-	-	2.8	-	-
44	ac-10	Safranal	1999	-	0.2	1.1	1.2	-	-	-	-
45	nt	Decanal	1205	5.7	0.5	1.2	0.8	0.6	1.0	0.5	0.5
46	om	Cumin aldehyde	1240	-	0.3	0.9	1.1	-	1.8	1.1	-
47	om	Carvone	1245	-	0.4	2.9	2.6	0.2	4.1	2.1	-
48	om	Thymoquinone	1250	-	-	-	-	-	0.8	-	-
49	om	Linalyl acetate	1258	-	0.3	0.5	-	0.3	0.1	-	0.8
50	nt	(*E*)-2-Decenal	1263	-	0.3	-	-	0.6	0.9	0.2	0.3

51	om	Isobornyl acetate	1286	-	0.5	0.6	0.4	0.2	0.7	0.4	0.3
52	pp	(*E*)-Anethole	1290	-	-	0.4	-	10.1	1.1	0.6	-
53	nt	Tridecane	1299	1.7	0.1	0.2	0.2	-	0.1	-	-
54	om	4-Terpinyl acetate	1300	-	-	-	-	-	-	-	0.1
55	om	Carvacrol	1302	-	-	-	-	-	0.5	1.2	-
56	nt	Undecanal	1312	2.5	-	-	-	-	-	-	-
57	nt	4-Vinyl guaiacol	1315	-	-	-	-	-	0.4	-	-
58	om	α-Terpinyl acetate	1350	8.0	0.8	2.0	1.0	0.3	1.0	0.6	0.3
59	pp	Eugenol	1360	-	-	-	-	-	0.6	-	-
60	sh	α-Ylangene	1372	-	0.1	0.2	0.3	-	-	0.2	0.5
61	sh	α-Copaene	1376	-	0.5	1.0	0.8	0.4	0.5	0.5	1.3
62	nt	Tetradecane	1399	3.8	0.3	0.5	1.8	-	0.4	0.2	0.5
63	sh	Isocaryophyllene	1404	-	0.3	0.3	0.7	0.4	1.0	0.3	0.1
64	nt	Dodecanal	1408	5.2	-	-	-	-	-	-	-
65	sh	β-Ylangene	1414	-	0.8	0.2	0.9	-	-	-	-
66	os	<4,8-α-epoxy> Caryophyllene	1417	-	-	0.2	0.6	-	-	-	-
67	sh	**β-Caryophyllene**	**1418**	**34.6**	**7.9**	**32.1**	**21.1**	**28.4**	**19.7**	**18.5**	**19.6**
68	sh	β-Deprezianene	1423	-	-	-	-	-	-	0.9	-
69	sh	γ-Elemene	1433	-	0.2	0.8	0.3	0.6	0.5	0.4	0.6
70	sh	β-Gurjunene	1435	-	-	-	-	-	0.7	-	-
71	sh	α-Guaiene	1442	-	-	-	-	-	0.2	-	-
72	sh	α-Himachalene	1448	-	0.3	0.5	0.6	-	2.0	0.5	0.1
73	ac-12	(*E*)-Geranyl acetone	1454	2.9	-	-	0.2	0.6	0.6	0.6	0.7
74	sh	α-Humulene	1455	1.2	0.6	2.1	1.4	2.2	1.8	1.3	1.1
75	sh	(E)-β-Farnesene	1458	-	0.2	-	0.3	-	-	-	-
76	nt	Dodecanol	1471	6.9		-	-	-	-	-	-
77	sh	γ-Muurolene	1477	0.2	0.3	0.7	0.6	-	0.5	0.4	-
78	sh	α-Amorphene	1480	-	-	-	-	0.1	-	-	0.3
79	sh	β-Selinene	1485	-	0.2	0.4	0.5	-	0.4	0.1	0.3
80	sh	Viridiflorene	1493	-	-	0.5	0.6	-	-	0.5	0.5
81	sh	*cis*-β-Guaiene	1494	-	-	-	-	-	0.2	-	-
82	sh	α-Muurolene	1499	-	-	0.4	0.1	1.1	-	0.6	1.1
83	nt	Pentadecane	1500	4.3	-	-	-	-	-	-	-
84	sh	β-Bisabolene	1509	-	-	-	0.2	-	0.2	0.1	-
85	sh	*trans*-γ-Cadinene	1513	-	-	0.7	-	0.5	-	-	0.7
86	sh	δ-Cadinene	1523	2.4	0.4	1.1	0.7	0.7	0.5	0.6	1.4
87	sh	α-Cadinene	1538	-	-	-	-	-	-	-	0.2
88	sh	Selina-3,7(11)- diene	1542	-	-	0.2	-	-	-	-	-
89	os	Elemol	1550	-	-	-	-	0.3	-	0.4	0.3
90	os	Caryophyllene alcohol	1570	-	-	0.2	0.2	-	0.3	0.2	-
91	os	Caryophyllene oxide	1581	-	-	-	-	-	-	-	0.6
92	nt	Hexadecene	1590	-	-	0.8	0.3	-	-	0.2	-
93	nt	Hexadecane	1600	-	0.3	0.2	0.2	-	-	-	0.5
94	sh	Cadalene	1674	-	-	-	0.3	-	0.3	0.2	0.2
95	nt	Isoamyl dodecanoate	1830	-	-	-	-	-	-	0.2	-
		**Total identified (%)**		**97.2**	**98.5**	**98.3**	**98.6**	**97.1**	**93.4**	**94.6**	**98.5**

	**Samples**	**1**	**2**	**3**	**4**	**5**	**6**	**7**	**8**
	**Class of compounds**								
mh	monoterpene hydrocarbons (mh)	-	62.8	34.6	32.5	34.7	19.7	43.5	59.6
om	oxygenated monoterpenes (om)	15.1	10.1	14.7	24.3	10.9	23.4	17.9	3.3
	**Total monoterpenes**	**15.1**	**72.9**	**49.3**	**56.8**	**45.6**	**43.1**	**61.4**	**62.9**
sh	sesquiterpenes hydrocarbons (sh)	38.5	11.8	41.2	29.4	35.5	28.3	25.1	28.0
os	oxygenated sesquiterpenes (os)	-	-	0.4	0.8	0.3	0.3	0.6	0.9
	**Total sesquiterpenes**	**38.5**	**11.8**	**41.6**	**30.2**	**35.8**	**28.6**	**25.7**	**28.9**
ac-10	apocarotenoids (ac-10)	-	0.2	1.1	1.2	-	-	-	-
ac-12	apocarotenoids (ac-12)	2.9	-	-	0.2	-	0.6	0.6	0.7
nt	non terpenes (nt)	40.7	8.2	5.9	10.2	5.6	16.6	6.3	6.0

pp	phenylpropanoids (pp)	-	-	0.4	-	10.1	4.5	0.6	-
	**Total identified (%)**	**97.2**	**98.5**	**98.3**	**98.6**	**97.1**	**93.4**	**94.6**	**98.5**

[a]LRI: Linear Retention Index on a DB5 column, [#]Samples of Sumac were collected from the different location and local market from Jordan. Sample 1 (Amman, Downtown), Sample 3 (Amman City), sample 4 (city Mall), Sample 5 (Al-Fuheis) and Sample 8 (Al-salt City). Sample 2 (Ajloun Forest Reserve), Sample 6 (Al-salt field) and Sample 7 (Al-Fuheis field).

Table S2. GC-MS analysis of Essential oil of *R. coriaria* fruits (Sumac samples 1-8) purchased from the Jordanian market and agriculture field.

				**Samples (relative content, %)[#]**							
				**EO-1**	**EO-2**	**EO-3**	**EO-4**	**EO-5**	**EO-6**	**EO-7**	**EO-8**
		**EO Yield (% w/w)**		**0.22**	**0.31**	**0.2**	**0.25**	**0.19**	**0.21**	**0.25**	**0.18**
**N°**	**Class**	**Compound**	**LRI [a]**								
**1**	**nt**	**Furfural**	**820**	**46.7**	**14.8**	**48.1**	**14.1**	**4.9**	**30.3**	**18.1**	**22.2**
2	nt	(*E*)-2-Hexenal	854	0.2	-	-	-	-	0.1	-	-
3	nt	5-methyl-2(3H)-Furanone	864	0.3	-	0.1	-	-	0.2	-	0.2
4	nt	2-Heptanone	890	0.1	-	-	-	-	-	-	-
5	nt	Cyclohexanecarboxaldehyde	896	0.2	-	-	0.1	-	-	-	-
6	nt	4-Nonene	897	-	-	-	-	-	0.1	-	-
7	nt	Heptanal	901	0.2	-	0.2	0.1	0.1	0.1	-	0.2
8	mh	*α*-Pinene	939	-	-	0.1	-	-	-	-	0.1
9	nt	1-ethyl-1-methyl-Cyclopentane	944	0.2	-	-	-	-	-	-	-
10	nt	3-propyl-cyclopentene	953	-	-	-	-	-	-	-	-
11	nt	(*E*)-2-Heptenal	955	5.1	0.1	1.3	0.1	0.1	1.4	0.1	0.4
12	nt	5-methyl-2-Furancarboxaldehyde	959	0.7	0.1	0.9	0.1	0	0.3	0.5	0.8
13	nt	Benzaldehyde	962	0.8	0.2	1.1	0.2	0.2	0.3	0.2	0.1
14	nt	Hexanoic acid	970	0.6	0.8	0.9	0.5	0.6	0.5	0.1	-
15	nt	1-Hepten-3-one	976	0.2	-	0.1	-	-	0.1	-	-
16	nt	1-Octen-3-ol	978	2.2	0.6	1.1	0.7	0.7	0.8	-	-
17	nt	6-methyl-5-Hepten-2-one	985	-	-	-	-	-	-	-	0.1
18	mh	Myrcene	991	-	-	0.1	-	-	-	-	-
19	nt	Mesitylene	995	-	-	0.1	-	-	-	-	-
20	nt	2-methyl-1,5-Hexadiene	997	0.1	-	-	-	-	-	-	-
21	nt	Decane	998	-	-	-	-	-	-	-	0.1
22	nt	Octanal	1002	0.2	0.1	0.3	0.1	0.1	0.2	0.1	0.3
23	mh	*α*-Phellandrene	1005	-	-	0.2	-	-	-	-	-
24	mh	δ-3-Carene	1011	0.2	-	0.9	-	-	-	-	-
25	mh	*α*-Terpinene	1018	-	0.1	-	-	0.1	-	-	0.2
26	mh	*p*-Cymene	1027	-	-	0.1	-	-	0.1	-	-
27	mh	Limonene	1031	-	0.1	1.1	-	-	0.2	0.1	0.4
28	om	1,8-Cineole	1035	0.1	0.3	0.6	0.3	0.4	0.4	0.2	0.2
29	nt	3-Octen-2-one	1036	0.1	0	0.1	-	-	-	-	-
30	nt	Phenylacetaldehyde	1044	0.1	0.1	0.8	0.1	0.2	0.4	0.3	0.4
31	nt	(*E*)-2-Octenal	1057	1.1	0.1	0.3	0.1	-	0.5	-	-
32	nt	1-Chloro-Octane	1061	-	-	-	-	-	0.1	-	-
33	nt	Cyclooctyl alcohol	1065	1.1	0.1	0.3	-	0.1	0.1	-	-
34	nt	Heptanoic acid	1067	-	0.3	-	0.1	0.3	-	-	0.1
35	nt	1-Octanol	1069	0.2	-	-	-	-	-	-	-
36	om	*cis*-Linalool oxide (furanoid)	1070	0.4	0.2	-	0.2	0.3	-	0.1	0.2
37	nt	3-Cyclohexene-1-methanol	1073	0.1	-	-	-	-	0.1	-	-
38	om	*trans*-Linalool oxide (furanoid)	1076	0.2	0.1	0.3	-	-	0.5	-	0.1
39	mh	Terpinolene	1088	-	-	-	-	-	-	0.2	0.3
40	om	Fenchone	1091	0.1	-	-	-	-	-	-	-
41	om	Linalool	1100	0.6	-	0.1	-	-	0.1	0.1	0.2
42	nt	Nonanal	1103	0.4	0.5	0.9	0.4	0.5	0.5	6.9	6.8
43	nt	2-Ethyl-hexanoic acid	1110	-	0.1	-	-	-	-	-	-
44	om	*allo*-Ocimene	1129	-	0.1	-	-	-	-	-	0.2
45	sh	Pinocarveol	1134	-	-	-	-	-	-	-	0.1
46	om	Terpinen-1-ol	1138	-	0.1	-	0.1	0.1	-	-	0.2
47	om	Camphor	1145	-	0.1	-	0.1	0.1	0.1	-	-
48	om	Menthone	1154	0.1	-	-	-	-	-	-	-
49	nt	(*E*)-2-Nonenal	1159	0.3	-	-	-	0.1	0.1	0.2	0.3
50	nt	Octanoic acid	1165	0.3	1.4	-	1.4	1.5	0.5	0.3	0.7
51	om	Borneol	1166	0.2	-	0.6	-	-	-	-	0.2
52	nt	Methyl 2-hydroxydodecanoate	1169	0.1	-	-	-	-	-	-	-

53	nt	1-Nonanol	1171	-	0.1	-	0.1	0.1	-	0.2	-
54	om	Menthol	1173	-	-	-	0.2	-	-	-	-
55	om	4-Terpineol	1180	0.6	0.5	0.2	0.7	0.7	0.5	0.2	-
56	om??	p-Cymen-8-ol	1188	0.1	-	-	-	-	-	-	-
57	om	**α-Terpineol**	**1190**	**1.8**	**1.4**	**1.4**	**1.6**	**1.9**	**1.5**	**3.0**	**2.4**
58	om	(+) Dihydrocarvone	1199	0.3	0.4	-	0.4	0.6	0.4	-	-
59	om	γ-Terpineol	1201	-	0.1	-	0.1	-	-	-	-
60	nt	Decanal	1205	-	0.1	-	-	-	-	0.2	0.2
61	nt	Octyl acetate	1208	-	-	-	-	-	-	-	-
62	nt	(E,*E*)-2,4-Nonadienal	1216	0.2	-	-	-	-	-	-	0.1
63	om	Pulegone	1240	-	0.1	-	-	-	-	-	-
64	om	**Carvone**	**1245**	**3.6**	**7.2**	**1.8**	**7.8**	**9.1**	**5.1**	**3.8**	**3.2**
65	nt	Phenylethyl acetate	1254	-	0.1	-	-	-	-	-	-
66	nt	Nonanoic acid	1262	-	0.8	-	-	-	-	2.1	3.8
67	nt	(E)-2-Decenal	1263	0.5	0.7	0.6	1.4	1.7	0.8	2.2	1.6
68	nt	(*E*)-Cinnamaldehyde	1276	8.5	-	0.8	-	-	2.6	-	-
69	om	Isobornyl acetate	1286	-	-	-	-	-	-	0.1	-
70	pp	(*E*)-Anethole	1290	4.1	-	-	3.4	3.9	3.1	1.3	-
71	nt	Phenylethanediol	1293	0.3	-	-	-	-	0.1	-	-
72	un	unidentified	1295	-	-	-	-	-	-	0.6	0.4
73	om	**Carvacrol**	**1302**	**7.7**	**6.9**	**3.2**	**3.9**	**4.3**	**5.0**	**1.0**	**1.8**
74	nt	Undecanal	1307	-	0.1	-	0.1	0.1	-	0.2	0.2
75	nt	*p*-Vinyl guaicol	1311	0.3	-	-	-	-	0.2	-	-
76	nt	(*E,E*)-2,4-Decadienal	1319	0.4	0.1	-	0.1	0.1	0.1	2.6	4.0
77	nt	2,9-Dimethyl-5-decyne	1333	-	-	-	0.2	-	-	-	-
78	nt	Methyl 11,14,17-eicosatrienoate	1334	0.1	0.2	-	-	0.1	0.1	-	-
79	nt	3-Nonen-2-one	1339	-	0.2	-	0.3	0.2	0.1	-	-
80	om	α-Terpinyl acetate	1350	0.2	1	-	1.1	1.3	0.6	0.5	0.4
81	nt	*n*-Decanoic acid	1359	0.1	0.5	-	0.4	0.5	-	-	0.2
82	pp	Eugenol	1360	2.9	0.8	0.5	0.8	0.9	1.5	-	-
83	nt	(E)-2-Dodecenal	1364	-	0.1	-	-	-	-	0.3	0.5
84	sh	α-Ylangene	1372	0.1	0.2	-	0.3	-	-	-	0.3
85	sh	Caryophyllene	1373	-	0.2	-	0.2	0.2	0.2	0.4	-
86	mh	Geranyl acetate	1375	-	0.1		0.1	0.1	-	-	0.5
87	sh	α-Copaene	1376	0.1	1.1	0.2	1.3	1.4	0.7	0.6	-
88	om	β-Damascenone	1380	0.1	-	-	-	-	-	-	-
89	sh	(-)-β-Elemene	1391	-	0.1	-	-	-	-	-	0.7
90	pp	Methyleugenol	1397	0.5	0.6	-	0.6	0.8	0.5	0.3	-
91	nt	Tetradecane	1399	-	0.1	0.3	0.1	0.1	-	0.3	0.2
92	sh	β-Ylangene	1414	-	-	-	0.1	0.1	-	0.2	-
93	os	<4,8-α-epoxy> Caryophyllene	1417	-	0.2	-	-	-	-	-	-
94	sh	**β-Caryophyllene**	**1418**	**1.2**	**7.5**	**6.7**	**8.7**	**10.1**	**4.5**	**10.6**	**8.1**
95	nt	1-Methyl-3-phenylallyl alcohol	1421	-	-	-	-	-	-	0.2	-
96	sh	β-Deprezianene	1423	-	0.1	-	-	0.1	-	-	-
97	sh	β-Copaene	1428	-	0.1	-	-	-	-	-	0.6
98	sh	γ-Elemene	1433	-	0.2	-	-	0.2	-	0.3	-
99	sh	*trans*-α-Bergamotene	1439	-	0.1	-	-	0.3	-	0.3	-
100	nt	5,9-Undecadien-2-one, 6,10-dimethyl-, (Z)-	1445	-	-	-	-	-	-	-	1.5
101	sh	α-Himachalene	1448	0.1	0.9	0.5	0.8	1.0	0.4	1.8	-
102	ac-12	(*E*)-Geranyl acetone	1454	-	0.1	0.2	-	-	-	-	-
103	sh	(+)-Valencene	1454	-	0.2	0.2	0.2	-	0.1	0.5	1.8
104	sh	α-Humulene	1455	-	1.1	0.7	0.8	1.1	0.5	1.2	1.2
105	sh	γ-Muurolene	1477	-	0.5	0.2	0.4	0.4	0.2	0.5	0.7
106	sh	α-Amorphene	1480	-	0.6	-	0.5	0.7	0.3	0.4	0.3
107	sh	Viridiflorene	1493	-	0.1	-	-	-	-	-	-
108	sh	*cis*-β-Guaiene	1494	-	0.9	-	0.5	0.6	0.2	0.3	-
109	sh	Bicylogermacrene	1494	-	-	-	-	-	-	-	0.2
110	sh	Aromandendrene	1496	-	-	0.2	-	-	-	-	-
111	sh	β-Cadinene	1501	-	0.5	0.3	0.7	0.7	0.3	0.4	0.2

112	nt	Butylated Hydroxytoluene	1503	0.2	-	-	0.1	0.1	-	-	-
113	nt	3,5-bis(1,1-dimethylethyl)-Phenol	1504	-	0.1	-	-	-	-	-	-
114	sh	β-Bisabolene	1509	0.2	0.4	0.3	0.5	0.6	0.3	0.8	-
115	sh	*trans*-γ-Cadinene	1513	-	0.2	0.1	0.3	0.1	-	0.3	0.5
116	nt	1,1,4,5,6-Pentamethyl-2,3-dihydro-1H-indene	1521	-	-	-	-	-	-	-	0.8
117	sh	δ-Cadinene	1523	-	0.9	0.6	1.1	1.1	0.5	2.1	-
118	sh	*trans*-Calamenene	1525	-	-	-	-	-	-	0.3	0.3
119	sh	β-Sesquiphellandrene	1525	-	0.4	-	0.5	0.5	0.2	-	-
120	sh	*α*-Cadinene	1538	0.1	0.2	-	0.1	0.1	-	-	-
121	sh	*α*-Calacorene	1542	-	0.2	-	0.2	0.2	-	0.2	0.2
122	nt	Dodecanoic acid	1555	-	1.2	-	1.1	1.2	0.3	0.2	-
123	sh	Acorenone B	1561	-	0.8	0.7	0.8	1.0	0.4	2.1	2.0
124	nt	Tetradecane, 1-iodo-	1568	-	0.1	-	-	-	-	-	0.5
125	os	Caryophyllene alcohol	1570	0.1	0.9	0.4	0.9	1.0	0.5	1.0	0.6
126	sh	(-)-Palustrol	1570	-	-	-	-	-	-	-	0.2
127	os	Caryophyllene oxide	1581	-	0.1	-	0.1	0.2	-	-	0.5
128	nt	Hexadecene	1590	-	0.2	0.1	0.2	0.2	-	0.3	-
129	nt	Hexadecane	1600	-	0.1	0.2	0.1	0.1	0.1	0.1	-
130	sh	Viridiflorol	1608	-	0.6	-	-	-	-	0.2	-
131	nt	Tetradecanal	1611	0.5	0.1	0.1	0.2	0.1	0.4	-	-
132	sh	γ-Eudesmol	1637	-	0.1	-	0.1	0.1	-	-	-
133	sh	*α*-Cadinol	1660	-	0.5	0.1	0.3	0.4	0.2	0.5	-
134	sh	*Ar*-Turmerone	1664	0.1	0.2	-	0.1	0.2	0.2	0.3	0.3
135	nt	Methyl tetradecanoate	1720	-	0.1	0.1	0.2	0.1	0.1	0.1	-
136	nt	Tetradecanoic acid	1753	-	0.4	-	0.2	0.3	0.1	-	0.3
137	nt	Octadecane	1796	-	-	-	0.1	0.1	0.1	0.1	0.2
138	nt	6,10,14-trimethyl-2-Pentadecanone	1838	-	0.4	0.2	0.4	0.4	0.3	1.0	-
139	nt	2-Pentadecanone, 6,10,14-trimethyl-	1840	-	-	-	-	-	-	-	1.2
140	nt	1,2-Benzenedicarboxylic acid, bis(2-methylpropyl) ester	1856	-	0.1	-	-	-	-	-	-
141	nt	2-Heptadecanone	1897	-	0.3	-	0.3	0.3	0.2	0.2	0.3
142	ac-12	Farnesyl acetone	1907	.	0.4	0.2	0.5	0.5	0.2	1.0	0.9
143	nt	Methyl palmitate	1920	-	2.6	1.4	3.1	3.4	1.6	1.7	1.3
144	dh	Cembrene	1931	0.2	6.1	8.0	7.3	8.4	4.1	10.7	7.4
145	nt	Palmitic acid	1955	-	9.0	2.6	8.8	9.4	6.6	3.8	2.9
146	nt	Ethyl palmitate	1988	-	0.2	0.2	0.3	0.2	0.1	0.1	-
147	ac-10	Safranal	1999	-	0.1	-	-	-	-	-	-
148	nt	Methyl linoleate	2088	-	1.5	0.6	1.7	1.8	0.8	1.4	-
149	nt	Methyl linolelaidate	2093	-	1.2	-	0.7	0.9	0.7	-	0.5
150	nt	Methyl elaidate	2095	-	3.5	4.4	4.2	4.7	1.9	2.3	0.8
151	nt	Heneicosane	2101	-	-	-	-	-	-	-	0.6
152	nt	Methyl stearate	2121	-	0.2	-	0.1	0.2	0.2	0.1	-
153	nt	Oleic Acid	2134	-	5.8	-	3.5	4.4	1.5	-	-
154	nt	Linoleic acid	2155	-	-	-	-	0.9	-	-	-
155	nt	Stearic acid	2156	-	0.1	-	-	-	-	-	-
156	nt	Ethyl Oleate	2162	-	0.1	-	-	-	-	-	-
157	nt	Linoelaidic acid	2170	-	-	-	-	-	-	-	0.3
158	nt	Hexadecanamide	2174	-	0.3	-	0.1	-	-	-	-
159	nt	*n*-Tricosane	2295	-	0.1	-	0.1	-	-	0.1	-
160	nt	Stearamide	2382	-	0.2	-	-	-	-	-	-
161	nt	*n*-Pentacosane	2494	-	-	-	-	-	-	0.4	-
162	nt	n-Hexacosane	2593	-	-	-	-	-	-	0.1	0.9
163	nt	n-heptacosane	2692	-	0.1	-	0.2	0.2	-	1.0	-
164	nt	Squalene	2806	-	0.1	-	-	-	0.2	-	-
165	nt	*n*-nonacosane	2892	-	-	-	-	-	-	0.5	-
		**Total identified (%)**		**98.7**	**97.1**	**98.8**	**94.7**	**96.7**	**89.3**	**95.5**	**92.4**
		**Class of compounds (%)**		**EO-1**	**EO-2**	**EO-3**	**EO-4**	**EO-5**	**EO-6**	**EO-7**	**EO-8**
	mh	monoterpene hydrocarbons (mh)		0.2	0.3	2.5	0.1	0.2	0.3	0.3	1.5

om	oxygenated monoterpenes (om)	16.1	18.5	8.2	16.5	18.8	14.2	9.0	9.1
	**Total monoterpenes**	**16.3**	**18.8**	**10.7**	**16.6**	**19**	**14.5**	**9.3**	**10.6**
sh	sesquiterpenes hydrocarbons (sh)	1.9	18.9	10.8	18.5	21.2	9.2	24.3	17.7
os	oxygenated sesquiterpenes (os)	0.1	1.2	0.4	1.0	1.2	0.5	1.0	1.1
	**Total sesquiterpenes**	**2.0**	**20.1**	**11.2**	**19.5**	**22.4**	**9.7**	**25.3**	**18.8**
dh	diterpene	0.2	6.1	8.0	7.3	8.4	4.1	10.7	7.4
ac-10	apocarotenoids (ac-10)	-	0.1	-	-	-	-	-	-
ac-12	apocarotenoids (ac-12)	-	0.1	0.2	-	-	-	-	-
nt	**non terpenes (nt)**	**72.7**	**50.5**	**68.2**	**46.5**	**41.3**	**55.9**	**48.6**	**55.6**
pp	phenylpropanoids (pp)	7.5	1.4	0.5	4.8	5.6	5.1	1.6	-
	**Total identified (%)**	**98.7**	**97.1**	**98.8**	**94.7**	**96.7**	**89.3**	**95.5**	**92.4**

[a] LRI: Linear Retention Index on a DB5 column (NIST-2017), #Samples of Sumac were collected from the different location and local market from Jordan. Sample 1 (Amman, Downtown), Sample 3 (Amman City), Sample 4 (city Mall), Sample 5 (Al-Fuheis) and Sample 8 (Al-salt City). Sample 2 (Ajloun Forest Reserve), Sample 6 (Al-Salt field) and Sample 7 (Al-Fuheis field).
